# Influence of Different Levels of Lipoic Acid Synthase Gene Expression on Diabetic Nephropathy

**DOI:** 10.1371/journal.pone.0163208

**Published:** 2016-10-05

**Authors:** Longquan Xu, Sylvia Hiller, Stephen Simington, Volker Nickeleit, Nobuyo Maeda, Leighton R. James, Xianwen Yi

**Affiliations:** 1 Department of Pathology and Laboratory Medicine, University of North Carolina, Chapel Hill, North Carolina, United States of America; 2 Division of Nephrology and Hypertension, Department of Medicine, University of Florida, Jacksonville, Florida, United States of America; University of Kentucky, UNITED STATES

## Abstract

Oxidative stress is implicated in the pathogenesis of diabetic nephropathy (DN) but outcomes of many clinical trials are controversial. To define the role of antioxidants in kidney protection during the development of diabetic nephropathy, we have generated a novel genetic antioxidant mouse model with over- or under-expression of lipoic acid synthase gene (*Lias*). These models have been mated with *Ins2*^*Akita/+*^ mice, a type I diabetic mouse model. We compare the major pathologic changes and oxidative stress status in two new strains of the mice with controls. Our results show that *Ins2*^*Akita/+*^ mice with under-expressed *Lias* gene, exhibit higher oxidative stress and more severe DN features (albuminuria, glomerular basement membrane thickening and mesangial matrix expansion). In contrast, *Ins2*^*Akita/+*^ mice with highly-expressed *Lias* gene display lower oxidative stress and less DN pathologic changes. Our study demonstrates that strengthening endogenous antioxidant capacity could be an effective strategy for prevention and treatment of DN.

## Introduction

Alpha-lipoid acid (1, 2-dithiolane-3-pentanoic acid, LA) is a potent antioxidant produced in mitochondria by lipoic acid synthase (LIAS) [1]. Among all natural antioxidants, LA plays a central role in the antioxidant network. It has several unique characteristics that include: 1) serving as a powerful antioxidant in both the oxidized and reduced forms; 2) quenching of a variety of reactive oxygen species (ROS); and 3) regenerating other antioxidants such as oxidized vitamins C and E, coenzyme Q10 and glutathione [2, 3]. LA is also a cofactor for several mitochondrial enzymes such as pyruvate dehydrogenase complex and α-ketoglutarate dehydrogenase complex, both of which participate in energy generation [4]. Complete lack of the *Lias* gene leads to mouse embryonic death, further underscoring the pivotal role LA plays in antioxidant defense and as a metabolic requirement [5].

Diabetic nephropathy (DN) is a leading cause of end-stage renal disease [6]. Oxidative stress has been suggested to play an important role in the development of DN [7, 8]. On the basis of epidemiological evidence, antioxidant therapy is a plausible strategy for treatment of this oxidative stress-related disease. However, there are conflicting reports regarding the effect of chronic dietary supplementation with antioxidants on outcomes of diabetic kidney disease in clinical trials [9–12]. An explanation for these observations is that natural genetic variation leads to inter-individual variations with respect to basic endogenous antioxidant levels. Indeed, it is well known that natural genetic variation affects gene expression levels and thus impacts molecular and physiological phenotypes such as protein expression levels. As a consequence, different endogenous antioxidant levels in tested individuals may influence experimental results and outcome of clinical trials aimed at examining the impact of antioxidant therapy on disease course. Thus, we hypothesize that the extent of oxidative damage is mainly dependent on endogenous antioxidant levels, especially in the initiation stage of diseases in which oxidative stress is implicated. To test our hypothesis, we have generated a group of unique lipoic acid hypomorphic and hypermorphic antioxidant mice by genetically modifying the 3’-untranslated region (3’-UTR) of lipoic acid synthase; this strategy is a similar approach that has been previously reported [13]. By comparing the major parameters of DN and oxidative stress in *Ins2*^*Akita/+*^ type 1 diabetic mice with different levels of endogenous *Lias* gene expression, we sought to define the role of oxidative stress in the onset and development of DN and obtain a better understanding of impact of antioxidants.

## Materials and Methods

### Creation of *Lias*^*Low/+*^ and *Lias*^*High/+*^ Mice by Changing 3’ untranslated region (3’-UTR) Sequences

The targeting construct prior to recombination consisted of a 3’-UTR of the endogenous *Lias* gene and was replaced after recombination with a cassette. The cassette consisted of the 3’-UTR sequences of bovine growth hormone gene (*bGH*) and a *Neo* gene, two lox P sites flanking the two fragments, and followed by the 3’-UTR of *cFos* gene. Colonies surviving after selection with G418 and ganciclovir were first screened by PCR with the following primers: a common primer (5′-CTA AAG TGT AGC CAA GCC CT-3′), a primer for screening *Lias* 3’-UTR (5′-CCT CCT CAG CTA CTG ACA TT-3′), a primer for bGH 3’-UTR (5′-GAG GCA AAC AAC AGA TGG CT-3’) and a primer for cFos 3’-UTR (5′-CTT CTC TGA CTG CAG ATC CT-3’). Targeted Embryonic stem (ES) cells were identified by the presence of approximately 200 bp PCR product for bGH 3’-UTR, and 300 bp after Cre recombinase-mediated recombination. Germline recombination was achieved using the B6.FVB-Tg (EIIa-Cre) stock (JAX#3724). These results were confirmed by Southern blot analysis.

The hypomorphic (*Lias*^*Low/Low*^) and hypermorphic (*Lias*^*High/High*^) *Lias* mice in C57BL/6 genetic background, with 25% or 150% of wildtype *Lias* gene expression respectively, were mated with C57BL/6-*Ins2*^*Akita/+*^ diabetic mice (JAX#3548), an established mouse model of type I diabetes mellitus [14, 15]. The *Ins2*^*Akita/+*^ mice have a mutation changing cysteine 96 to tyrosine in the insulin 2 gene and exhibit marked hyperglycemia as early as 4 weeks of age [15]. Eight B6-*Lias*^*Low/Low*^*Ins2*^*Akita/+*^ males and 9 B6-*Lias*^*High/High*^*Ins2*^*Akita/+*^ males were obtained from crossing C57BL/6-*Ins2*^*Akita/+*^ female mice and *Lias*^*Low/Low*^ or *Lias*^*High/High*^ male mice. Only males were phenotyped in this study because *Ins2*^*Akita/+*^ females on the B6 genetic background displayed much less severe diabetic phenotype than the males [16]. *Lias*^*+/+*^*Ins2*^*Akita/+*^ mice served as a control. In addition, the mice were fed normal mouse chow (Research Diets, Inc. New Brunswick, NJ) and had *ad libitum* access to autoclaved water. All animal protocols were approved by the University of North Carolina at Chapel Hill Institutional Animal Care and Use Committee (Protocol numbers: 13-208-0 and16-153-0).

### Biochemical Parameters

Blood glucose was monitored monthly in the mice from 7 to 28 weeks using the One-Touch Lifescan meter (Lifescan Inc, Milpitas, CA) on samples obtained after a 5-hour fasting period. Plasma glucose, total cholesterol and triglyceride were examined using assay kits (Wako, Richmond, VA). Lactic acid concentration in tissues was determined as described [17]. Pyruvate concentration was measured using a pyruvate assay kit (BioVision, Mountain View, CA).

### Urinary Albumin Excretion

At 28 weeks of age, individual mice were placed in metabolic cages to record food consumption, water intake, body weight, and urine output for 48 hours prior to sacrifice. Urine albumin in *Lias*^*High/High*^ *Ins2*^*Akita/+*^ and *Lias*^*Low/Low*^*Ins2*^*Akita/+*^ mice was assessed by ELISA using Albuwell according to the manufacturer’s instructions (Exocell, Philadelphia, PA). Urinary creatinine levels were determined by the Creatinine Kit (Exocell) according to the manufacturer’s instructions.

### Urinary MCP-1 assay

Urinary monocyte chemoattractant protein-1 (MCP-1) excretion was measured as markers of renal inflammation. The MCP-1 concentration was measured using an ELISA assay kit (Research & Diagnostic Systems, Minneapolis, MN) according to the manufacturer’s instructions. The ELISA kit was specific for mouse MCP-1 and sensitive down to 2 pg/ml. The MCP-1 concentration was normalized to the urinary creatinine concentration.

### Blood Pressure Measurement

Systolic blood pressure (BP) was determined in conscious mice using a tail-cuff method [18]. The first 10 readings were discarded, and 30 readings were taken to obtain daily BP. Average BP on 5 consecutive days was taken to represent BP of each mouse.

### Renal Function and Morphometric Analyses

Mice were perfused at 120 mmHg with 0.9% saline containing heparin, followed by 4% paraformaldehyde and then embedded in paraffin and 3-μm sections were cut. Sections were stained with hematoxylin and eosin (H&E) and Periodic acid-Schiff's base (PAS) for examination by light microscopy. Mesangial matrix expansion (MME) was examined in a blinded fashion and scored from 0 to 4 according to the ratio of glomerular expansion area/normal area: score 0, a normal glomerulus; score 1, increased mesangial matrix <25% of glomerular tuft; score 2, MME of 25%–50% of glomerular tuft; score 3, MME of 50%–75%; and score 4, MME of >75% of the tuft. MME was derived from assessment of three glomerular profiles on each mouse. Next, the kidney cortex was conventionally prepared for transmission electron microscopy. The samples were examined with an electron microscope (Model LEO 910; LEO Electron Microscopy Inc, Thornwood, NY). Three to four kidney samples from each experimental group were randomly chosen for electron microscopic observation. Thickness of glomerular basement membranes (GBM) was measured from transmission electron microscopy photos (magnification, x12,000) at 5 specific points along each of 10 randomly selected glomerular capillaries to determine an average GBM thickness for 5 mice in each group. Mitochondrial damage in proximal tubules was examined in each genotypic group. Briefly, under the same magnification (x10,000), about 200–300 mitochondria from 10–12 randomly selected fields in each genotypic group were observed. The Degree of the mitochondrial damage was assessed according to the following scale from 0 to 3: 0) indicating a normal structure, 1) normal with slight swelling, 2) mitochondrial swelling and cristae dilated/disorder, and 3) mitochondrial vacuolization. On the basis of the above criteria, the degree of mitochondrial damage in the different groups of mice were scored and the total scores of mitochondria per group were summarized and then divided by total counted mitochondrion number in each group to get the ratio of damaged over total counted mitochondrion number. The ratio indicates the degree of mitochondrial damage.

### Assessment of Oxidative Stress

To evaluate oxidative stress, the concentration of urinary 8-isoprostane was measured using an enzyme immunoassay and expressed relative to the level of urine creatinine following the manufacturer’s protocol (Cayman Chemical Inc., Ann Arbor, MI). Systemic oxidative stress in blood was determined using 4-Hydroxynonenal (4-HNE) assay kit in accordance with manufacturer’s specifications (Cell Biolabs, Inc. San Diego, CA).

### Western Blot Analysis

Total protein was extracted from the renal cortical tissues with RIPA buffer and protein concentration was determined by BCA protein assay method (Thermo Scientific, Rockford, IL) following manufacturer’s instructions. Western blot analysis was performed using a rabbit polyclonal antibody against mouse LIAS (GeneTex, Inc.Irvine, CA), and (voltage-dependent anion-selective channel protein 1) VDAC1 as mitochondrial loading control (Abcam, Cambridge, MA) and the protein bands were quantified with Image Quant LAS4000 software (GE Healthcare, Piscataway, NJ).

### Reverse Transcription and Quantitative Real-Time Polymerase Chain Reaction (RT-PCR)

Total RNA was extracted from kidney cortex using an ABI 6700 Automated Nucleic Acid Workstation following the manufacturer’s protocol (Applied Biosystems, Foster City, CA). Relative mRNA amounts were determined using real-time quantitative reverse transcriptase-PCR (Applied Biosystems) with *β-*actin as the reference gene in each reaction [18]. The expression of the genes *Lias*, superoxide dismutase 2 *(Sod2)*, transforming growth factor β1 (*Tgfβ1)*, nuclear factor (erythroid-derived 2)-like 2 (*Nrf2*) and NADPH oxidase 4 (*Nox4*) were examined.

### Statistical Analyses

Data were expressed as mean ± standard error of the mean (SEM). *P*<0.05 was considered significant. *P* values are obtained for comparisons among *Lias*^*Low/Low*^*Ins2*^*Akita//+*^, *Lias*^*High/High*^*Ins2*^*Akita/+*^ and *Lias*^*+/+*^*Ins2*^*Akita/+*^ mice using one-way or two-way ANOVA. Post hoc pairwise comparisons were performed by Tukey–Kramer honestly significant differences (HSD) test (JUM, SAS Institute, Cary, NC).

## Results

### Model Construction

**Fig 1:**
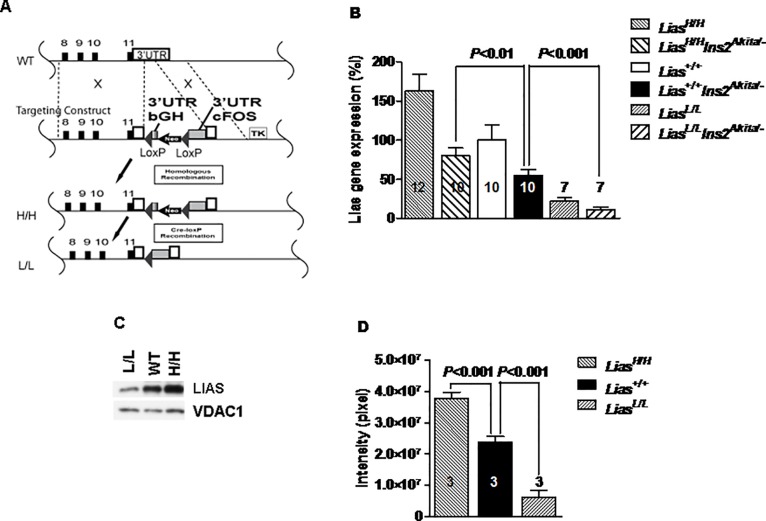
Characterizations of the new mouse model. (A) Generation of mice with genetically graded expression of lipoic acid synthase (*Lias*). Wild type (top line): Endogenous *Lias* gene 3’-UTR (white column) is located after exon 11 of *Lias* gene. The targeting construct (second line): consisted of the 3’-UTR sequences of bovine growth hormone (*bGH*) gene and a *Neo* gene, two lox P sites flanking the two fragments, and followed by the 3’-UTR of *cFos* gene and thymidine kinase gene (TK). H/H (third line): The locus after homologous recombination. *Lias* expression is now controlled by the 3’-UTR of *bGH*, which stabilizes *Lias* mRNA. L/L (bottom line): The locus after Cre-lox P recombination. *Lias* expression is controlled by the 3-UTR of *cFos*, which destabilizes the *Lias* mRNA. (B) The kidney mRNA levels of *Lias* in 12-week-old L/L, H/H and WT male mice and in 28-week-old diabetic L/L, H/H and WT male mice. *Lias* gene expression in the non-diabetic WT mice as a reference for the all six groups of mice. (C) Lipoic acid synthase (LIAS) concentrations of kidney cortex mitochondria, measured by Western blot, and VDAC1 as loading control, in 12-week-old L/L, H/H and WT male mice. n = 5, in each group. (D) The amounts of lipoic acid in non-diabetic *Lias*^*High/High*^ and *Lias*^*Low/Low*^ kidney, detected by Western blot, were quantified by Image Quant software. Data are expressed as the mean ± SE.

As described in methods, the gene targeting strategy for engineering the hyper- and hypomorphic *Lias* mice with over- or under-expression of the *Lias* gene was based on genetic modification of the 3’-UTR (Fig 1A). The DNA sequence result showed that the orientation of the 3'UTR sequences of both *bGH* and *c-Fos* are replaced in the transcriptional orientation (data not shown). ES cell lines with a correctly targeted 3’-UTR were identified and injected into C57BL/6J recipient blastocysts, which were transferred to the uteri of CD-1 pseudopregnant dams. These mice initially produced stabilized transcripts of the *Lias* gene using the 3’-UTR sequence of *bGH*, but were changed to unstable transcripts using the 3’-UTR from the *cFos* gene after Cre-LoxP recombination was induced. The *Lias*^*High/+*^ mice were crossed with Tg (Ella-cre) mice that expressed Cre-recombinase in testis and thus the homozygous offspring (*Lias*^*Low/Low*^) generated from these *Lias*^*Low/+*^ founder males expressed low levels of *Lias*. Since plasma and tissue LA were not directly measured due to a technical difficulty [19], changes in organ LA levels were inferred by demonstrating changes in *Lias* gene expression using RT-PCR (Fig 1B) or Western blot (Fig 1C). Western blot results for kidney LIAS, quantitatively assessed by densitometry, showed that LIAS protein concentrations were about 150% in *Lias*^*High/High*^ mice and around 25% in *Lias*^*Low/low*^ mice, compared with those in *Lias*^*+/+*^ mice (Fig 1D).

### Systemic Pathological Changes

The hypomorphic (*Lias*^*Low/Low*^) and hypermorphic (*Lias*^*High/High*^) *Lias* mice, with 25% or 150% of wildtype *Lias* gene expression, respectively, were mated with *Ins2*^*Akita/+*^ diabetic mice, an established mouse model of diabetes mellitus that can mimic early stages of DN [14, 15]. *Lias*^*High/High*^ *Ins2*^*Akita/+*^ and *Lias*^*Low/Low*^*Ins2*^*Akita/+*^ mice exhibited the different diabetic phenotypes. *Lias*^*Low/Low*^*Ins2*^*Akita/+*^ mice had significantly lower body weight compared to both *Lias*^*High/High*^*Ins2*^*Akita/+*^ and *Lias*^*+/+*^*Ins2*^*Akita/+*^ mice as shown in Table 1 (*P*<0.05). Both *Lias*^*High/High*^*Ins2*^*Akita/+*^ and *Lias*^*Low/Low*^*Ins2*^*Akita//+*^ mice manifested hyperglycemia at 28 weeks of age but there was no significant difference between the two groups of mice. In addition, plasma total cholesterol and triglyceride levels were similar among *Lias*^*High/High*^*Ins2*^*Akita/+*^, *Lias*^*Low/Low*^*Ins2*^*Akita/+*^, and *Lias*^*+/+*^*Ins2*^*Akita/+*^ mice (Table 1).

**Table 1:** Laboratory data in experimental animals.

Parameters	*Lias*^*H/H*^*Ins2*^*Akita/+*^ n = 9	*Lias*^*+/+*^*Ins2*^*Akita/+*^n = 10	*Lias*^*L/L*^*Ins2*^*Akita/+*^ n = 8	*P* Value (ANOVA)
Systolic pressure (mmHg)	106±9	113±8	119±12	0.18
Diet intake (g/day)	8.4±0.6	7.6±0.6	9.4±0.8	0.27
Water intake (ml/day)	23.5±0.6	23.8±1.3	26.5±0.8	0.24
Body weight (BW, g)	29.6±1.1	26.7±1.1	22.6±1.5	<0.05
Plasma				
Glucose (mg/dl)	554±17	544±15	529±21	0.55
Cholesterol (mg/dl)	64±13	78±7	84±13	0.60
Triglyceride (mg/dl)	52±5	55±9	63±13	0.47
Kidney				
Urine volume (ml/day)	21.5±8.0	22.5±4.2	25.3±6.7	0.33
Kidney weight (KW, mg)	230±16	250±14	260±17	0.25
KW/BW (mg/g)	8.3±1.4	9.3±1.3	11.5±0.9	0.13
lactate (mM/mg protein)	0.87±0.10	1.14±0.11	1.26±0.18	0.09
pyruvate (mM/mg protein)	2.6±0.31	2.6±0.22	2.2±0.28	0.85

Data shown are mean values ±SEM for the male mice at 28 weeks.

*P* values are for comparisons among *Lias*^*Low/Low*^*Ins2*^*Akita//+*^, *Lias*^*High/High*^*Ins2*^*Akita/+*^ mice and *Lias*^*+/+*^*Ins2*^*Akita/+*^ mice using one way ANOVA.

### Kidney Pathologic Changes

**Fig 2:**
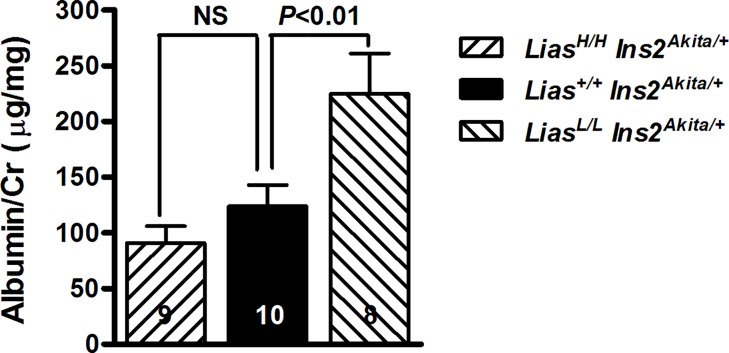
Changes of urine albumin in the mice. Urine albumin/creatinine ratio in *Lias*^*Low/Low*^*Ins2*^*Akita//+*^ mice is significantly elevated (*P*<0.01) whereas the ratio in *Lias*^*High/High*^*Ins2*^*Akita/+*^ mice is reduced but does not reach significance, compared with *Lias*^*+/+*^*Ins2*^*Akita/+*^ mice at 28 weeks of age. The numbers inside the bars indicate the number of animals.

Urinary albumin/creatinine ratio in *Lias*^*Low/Low*^*Ins2*^*Akita/+*^ mice was 2.6-fold higher than *Lias*^*+/+*^*Ins2*^*Akita/+*^ mice at 28 weeks of age whereas, the ratio in *Lias*^*High/High*^*Ins2*^*Akita/+*^ mice was about 30% lower than *Lias*^*+/+*^*Ins2*^*Akita*^ mice but was not statistically significant (Fig 2). Dietary intake and water consumption in *Lias*^*Low/Low*^*Ins2*^*Akita/+*^ and *Lias*^*High/High*^*Ins2*^*Akita/+*^ mice were not significantly different compared with *Lias*^*+/+*^*Ins2*^*Akita*^ mice (Table 1). The ratio of kidney weight-to-body weight (KW/BW) in *Lias*^*Low/Low*^*Ins2*^*Akita/+*^ mice was higher than control *Lias*^*+/+*^*Ins2*^*Akita/+*^ mice whereas this ratio was lower in *Lias*^*High/High*^*Ins2*^*Akita/+*^ mice than in control *Lias*^*+/+*^*Ins2*^*Akita/+*^ mice. Kidney levels of lactate and pyruvate were similar in all three diabetic groups (Table 1).

**Fig 3:**
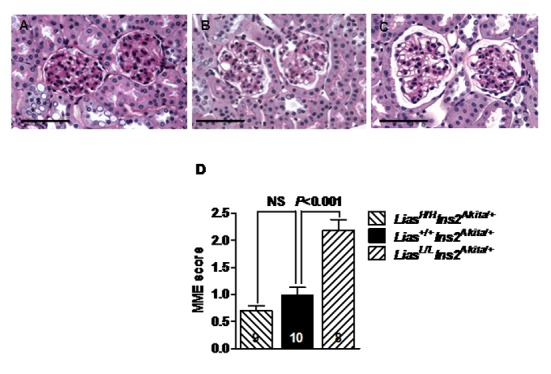
Changes of *Lias* gene expression influences mesangial matrix expansion (MME). Representative PAS staining of glomeruli in diabetic *Ins2*^*Akita//+*^ mice at 28 weeks of age. (A) *Lias*^*Low/Low*^*Ins2*^*Akita//+*^ mice. (B) *Lias*^*+/+*^*Ins2*^*Akita/+*^ mice. (C) *Lias*^*High/High*^*Ins2*^*Akita/+*^ mice. Original magnification x400, Bars = 50μm. (D) MME score, quantified as the region of positive PAS staining, is expressed as a function of total glomerular tuft area. The numbers inside the bars indicate the number of animals. *Lias*^*Low/Low*^*Ins2*^*Akita//+*^ and *Lias*^*High/High*^*Ins2*^*Akita/+*^ mice are compared with *Lias*^*+/+*^*Ins2*^*Akita/+*^ mice. Values are expressed as the mean ± SEM.

**Fig 4:**
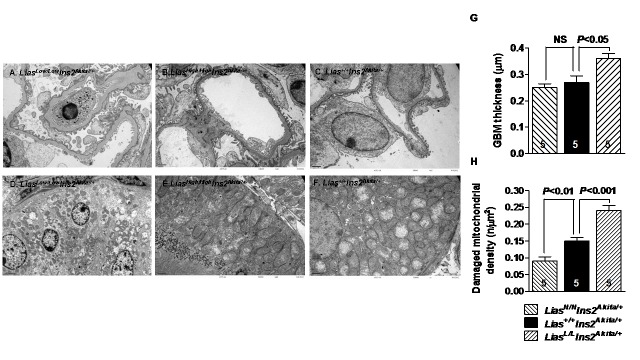
Representative electron micrographs from diabetic *Ins2*^*Akita//+*^ mice at 28 weeks of age. (A) Segmentally thickened glomerular basement membrane (GBM) with minor irregularities along the lamina rara externa (“undulations”) and segmental podocyte foot process effacement in *Lias*^*Low/Low*^*Ins2*^*Akita//+*^ mice. Original magnification, x8,000. (B) *Lias*^*High/High*^*Ins2*^*Akita/+*^ mice. Original magnification, x8,000. (C) *Lias*^*+/+*^*Ins2*^*Akita/+*^ mice. Original magnification, x8,000. (D) Illustrated is the basal part of a tubular epithelial cell containing distorted mitochondria. Cristae are disrupted and mitochondria filled presumably with lipid products vaguely resembling zebra-bodies in *Lias*^*Low/Low*^*Ins2*^*Akita//+*^ mice. Original Magnification, x5,000. (E) A small number of damaged mitochondria in proximal tubular cells of *Lias*^*High/High*^*Ins2*^*Akita/+*^ mice. Original magnification, x8,000. (F) Damaged mitochondria in proximal tubular cells of *Lias*^*+/+*^*Ins2*^*Akita/+*^ mice. Magnification, x8,000. (G) Electron microscopic quantitative examination using Image J showed thickening of the GBM significant increase in *Lias*^*Low/Low*^*Ins2*^*Akita/+*^ mice compared to *Lias*^*+/+*^*Ins2*^*Akita/+*^ mice. (H) Ratio of average damaged mitochondrion over entire counted mitochondria in proximal tubules of *Lias*^*Low/Low*^*Ins2*^*Akita//+*^, *Lias*^*High/High*^*Ins2*^*Akita/+*^ and *Lias*^*+/+*^*Ins2*^*Akita/+*^ mice, using one-way ANOVA for the comparison.

Renal pathological changes were assessed by light and transmission electron microscopy. Moderate mesangial matrix expansion, as evidenced by increased accumulation of PAS positive material in the mesangial area, was observed in 28-week-old *Lias*^*Low/Low*^*Ins2*^*Akita/+*^ mice (Fig 3A), whereas mesangial expansion was milder in *Lias*^*High/High*^*Ins2*^*Akita/+*^ mice (Fig 3C), compared to the *Lias*^*+/+*^*Ins2*^*Akita*^ control (Fig 3B). Semi-quantitative analysis of PAS-stained kidney sections revealed a higher mesangial expansion score (*P*<0.05) in *Lias*^*Low/Low*^*Ins2*^*Akita/+*^ mice as compared with *Lias*^*High/High*^*Ins2*^*Akita/+*^ and *Lias*^*+/+*^*Ins2*^*Akita/+*^ mice (Fig 3D). Electron microscopic examination showed thickening of the GBM in *Lias*^*Low/Low*^*Ins2*^*Akita/+*^ mice (Fig 4A), compared to *Lias*^*+/+*^*Ins2*^*Akita/+*^ mice (Fig 4C). Quantitative examination using Image J showed the thickening of the GBM significantly increased (0.36 ± 0.04 μm versus 0.27 ± 0.04 μm in, *P*<0.05, Fig 4G). Foot process effacement was primarily detected in *Lias*^*Low/Low*^*Ins2*^*Akita/+*^ mice (Fig 4A) but podocyte slit pore width did not exhibit significant differences among three groups of the mice. Considerable numbers of mitochondria within proximal tubules in *Lias*^*Low/Low*^*Ins2*^*Akita/+*^ mice were damaged as revealed by mitochondrial structural irregularities with swelling, disruption of cisternae and vacuolization. In contrast, few damaged mitochondria were observed in *Lias*^*High/High*^*Ins2*^*Akita/+*^ mice (Fig 4B and 4E) compared with *Lias*^*Low/Low*^*Ins2*^*Akita/+*^ and *Lias*^*+/+*^*Ins2*^*Akita/+*^ mice (Fig 4D and 4F). Damaged mitochondria were quantified in *Lias*^*High/High*^*Ins2*^*Akita/+*^ and *Lias*^*+/+*^*Ins2*^*Akita/+*^ mice at 28 weeks of age. As shown in Fig 4H, the average number of damaged mitochondria in proximal tubules of *Lias*^*Low/Low*^*Ins2*^*Akita/+*^ mice were significantly increased whereas damaged mitochondria in *Lias*^*High/High*^*Ins2*^*Akita/+*^ mice were significantly decreased compared to *Lias*^*+/+*^*Ins2*^*Akita/+*^ mice. Glomerulosclerosis, arteriolar hyalinosis and focal tubulointerstitial fibrosis were not detectable in all diabetic *Ins2*^*Akita/+*^ mice at 28 weeks of age regardless of *Lias* transcript level. In addition, no electron dense deposits were observed in the glomeruli in all mice.

### Oxidative Stress and Inflammation

**Fig 5:**
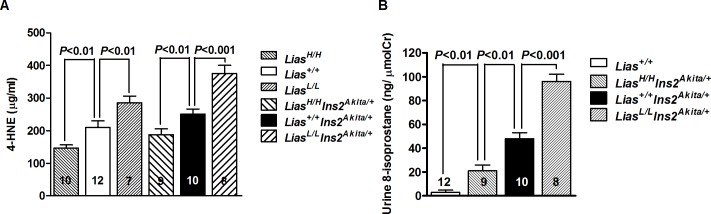
Alternated oxidative stress. (A). Oxidative stress in kidney cortex is associated with plasma concentration of lipid perioxidation marker, 4-hydroxynonenal (4-HNE), in diabetic *Ins2*^*Akita//+*^ mice and non-diabetic mice with differential *Lias* gene expression. *Lias*^*+/+*^ mice as a control. Data were analyzed using two-way ANOVA. (B). Urine 8-isoprostane varies with *Lias* expression in diabetic mice. Data were analyzed using one-way ANOVA. The numbers inside the bars indicate the number of animals in each group. Results are expressed as mean ±SEM.

Systemic oxidative stress, assessed by measuring 4-HNE, a well-known product of lipid peroxidation and a measure of oxidative stress, was significantly different in *Lias*^*High/High*^*Ins2*^*Akita/+*^ and *Lias*^*+/+*^*Ins2*^*Akita/+*^ mice compared with *Lias*^*+/+*^*Ins2*^*Akita/+*^ mice (Fig 5A). Another reliable lipid peroxidation marker, 8-isoprostane, was examined in urine. *Lias*^*Low/Low*^*Ins2*^*Akita/+*^ mice at 28-weeks-old showed noticeably higher levels than *Lias*^*+/+*^*Ins2*^*Akita/+*^ mice (Fig 5B). On the contrary, *Lias*^*High/High*^*Ins2*^*Akita/+*^ mice manifested significantly lower urinary 8-isoprostane levels than *Lias*^*+/+*^*Ins2*^*Akita/+*^ mice (Fig 5B).

Consistent with Western blot results for LIAS protein, RT-PCR analysis of kidney cortex showed significantly increased *Lias* gene expression in *Lias*^*High/High*^*Ins2*^*Akita/+*^ and significantly reduced expression in *Lias*^*Low/Low*^*Ins2*^*Akita/+*^ mice at 28 weeks of age (Table 2). Interestingly, gene expression of mitochondrial *Sod2*, a major antioxidant enzyme which responds to enhanced superoxide production in mitochondria, was significantly higher in *Lias*^*Low/Low*^*Ins2*^*Akita/+*^ mice, likely reflecting an attempt to compensate for reduced LIAS-generated LA in *Lias*^*Low/Low*^*Ins2*^*Akita/+*^ kidney. The data confirm our previous observation that *Sod2* expression also markedly increases in *Lias*^*+/-*^*Ins2*^*Akita/+*^ mice [20]. To identify the ROS sources, we examined *Nox4* gene expression in kidney cortex. Our result showed that *Nox4* gene expression was significantly increased at 28-weeks of age in *Lias*^*Low/Low*^*Ins2*^*Akita/+*^ whereas a significant decrease in *Lias*^*High/High*^*Ins2*^*Akita/+*^ kidney suggests that NADPH oxidase is a target for the antioxidant effects of lipoic acid (Table 2). In addition, significantly reduced *Tgfβ1* gene expression in *Lias*^*High/High*^*Ins2*^*Akita*^ mice (Table 2) suggests that increased *Lias* gene expression likely attenuates adverse effect(s) of TGF-β1.

**Table 2:** Gene expression in kidney cortex.

	*Lias*^*H*^*/*^*H*^*Ins2*^*Akita/-*^n = 10	*Lias*^*+*^*/*^*+*^*Ins2*^*Akita/-*^n *=* 10	*Lias*^*L*^*/*^*L*^*Ins2*^*Akita/-*^n *=* 7
*Lias*	1.39±0.23^aa^	1.00±0.14	0.19±0.05^aa^
*Sod2*	1.05±0.25	1.00±0.16	1.65±0.21^a^
*Tgfβ1*	0.67±0.16^a^	1.00±0.11	1.32±0.19
*Nrf2*	1.23±0.22	1.00±0.15	0.86±0.2
*Nox4*	0.57±0.15^a^	1.00±0.16	1.94±0.26^aa^

Samples were taken at 7 months of age of the mice. Data shown are mean values ±SEM using t-test.

a: *P*<0.05 vs *Lias*^*+*^*/*^*+*^*Ins2*^*Akita/+*^ mice.

aa: *P*<0.01 vs *Lias*^*+*^*/*^*+*^*Ins2*^*Akita/+*^ mice mRNA levels of *Lias*^*+*^*/*^*+*^*Ins2*^*Akita/+*^ mice were used as references and set to 1.00

NRF2 is a key transcription factor for regulation of antioxidant defense [21] and LA has been shown to stimulate NRF2 nuclear accumulation [22]. To test whether endogenous LA can affect NRF2, we examined *Nrf2* gene expression in 28-week-old *Lias*^*Low/Low*^*Ins2*^*Akita/+*^ and *Lias*^*High/High*^*Ins2*^*Akita/+*^ mouse kidney. Unlike *Sod2*, *Nox4* and *Tgfb1* transcripts, no significant changes in *Nrf2* expression were observed in response to *Lias* gene manipulation (Table 2).

**Fig 6:**
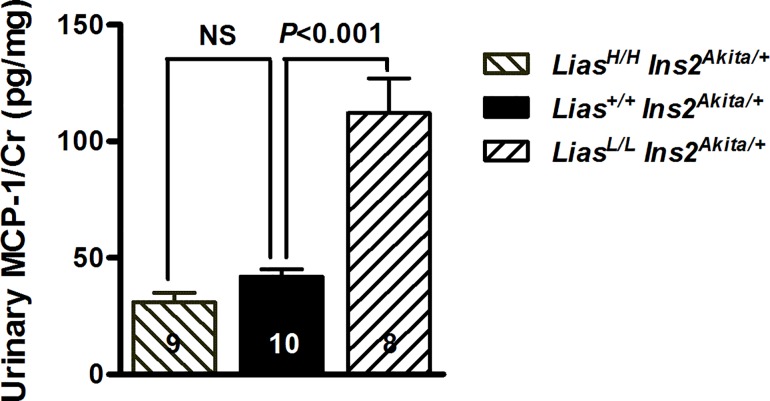
Kideny inflammation. Urinary MCP-1 levels in *Lias*^*Low/Low*^*Ins2*^*Akita/+*^, *Lias*^*+/+*^*Ins2*^*Akita/+*^ and *Lias*^*High/High*^*Ins2*^*Akita/+*^ mice. The numbers inside the bars indicate the number of animals in each group. Results are expressed as mean ±SEM.

Inflammation is thought to be a pathogenic factor in the initiation of DN and monocyte chemoattractant protein-1 (MCP-1) is considered as a major mediator of inflammation in DN patients. To further probe the underlying mechanism(s) of endogenous oxidant injury in relation to inflammation in diabetes mellitus, we measured urinary MCP-1. Our results showed that there were significantly increased urinary MCP-1 levels in *Lias*^*Low/Low*^*Ins2*^*Akita/+*^ mice compared with *Lias*^*+/+*^*Ins2*^*Akita/+*^ and *Lias*^*High/High*^*Ins2*^*Akita/+*^ mice (Fig 6).

## Discussion

In our previous study, the pathologic changes of diabetic nephropathy (DN) were exacerbated in *Lias*^*+/-*^*Ins2*^*Akita/+*^ mice with an approximately 50% reduced *Lias* gene expression, and the aforementioned pathology was associated with significantly enhanced oxidative stress [20]. To verify the central role of LIAS in generating endogenous LA for antioxidant defenses, we manipulated *Lias* transcript stability to generate two lines of novel antioxidant mouse models. In one, *Lias* gene expression was reduced to roughly 25% of normal, a level sufficient to maintain viability (*Lias* null mice are embryonic lethal). In the other, *Lias* gene expression was increased to approximately 150% of normal expression level, which is anticipated to afford better protection, than wildtype mice, against oxidative stress. When combined with the diabetogenic *Ins2*^*Akita*^ mutation, the double mutants allow evaluation of the impact of two different antioxidant baselines (Low or High) on the development of DN. As hypothesized, the new diabetic models exhibited divergent renal responses to diabetogenic stress when compared with *Lias*^*+/+*^*Ins2*^*Akita/+*^ mice. Major DN pathological changes such as albuminuria and mesangial expansion in *Lias*^*Low/Low*^*Ins2*^*Akita/+*^ mice were significantly worse than those in *Lias*^*+/+*^*Ins2*^*Akita/+*^ mice whereas the two pathological changes were less severe in *Lias*^*High/High*^*Ins2*^*Akita/+*^ mice. The data further support the correlation between *Lias* transcript abundance and inferred endogenous LA concentration; levels of two reliable common oxidative stress biomarkers, plasma 4-NHE and urine 8-isoprostane, were both significantly higher in *Lias*^*Low/Low*^*Ins2*^*Akita/+*^ mice and significantly lower in *Lias*^*High/High*^*Ins2*^*Akita/+*^ mice compared to *Lias*^*+/+*^*Ins2*^*Akita/+*^ controls. These results are consistent with our previous data where we used different lipid peroxidation markers, like TBARS and, antioxidant marker such as ratio of GSH/GSSG to reveal significantly increased oxidative stress and decreased endogenous antioxidant capacity in *Lias*^*+/-*^*Ins2*^*Akita/+*^ mice. Since lipoic acid plays a central role in the antioxidant network, the significantly increased oxidative stress and decreased endogenous antioxidant capacity observed in *Lias*^*+/-*^*Ins2*^*Akita/+*^ mice were very likely due to the impairment of the antioxidant defense system. In addition, our data demonstrate that *Lias* overexpression effectively attenuates albuminuria and kidney disorders without exerting a significant hypoglycemic effect in *Lias*^*High/High*^*Ins2*^*Akita/+*^ mice, suggesting that the protective effect of LA results primarily from its antioxidant capacity rather than from a direct hypoglycemic effect. These findings confirm that LIAS-generated lipoic acid plays a vital role in the early development of DN by demonstrating that diabetic mice with low endogenous antioxidant capacity manifestly increased ROS-mediated renal stress. In particular, the new models provide very strong proof-of-principle that an increased antioxidant reservoir could represent a powerful new tool in the prevention and / or treatment of diabetic kidney disease. A novel finding obtained from our current study underlines the importance of antioxidants for mitochondrial protection in the retardation of DN. Growing evidence indicates that mitochondria play a critical role in the initiation and development of DN. Mitochondria are believed to be the major organelles involved in superoxide generation. They consume approximately 85% of the oxygen used by cells and overproduction of superoxide anions may occur by excessive electron leak in the mitochondrial electron transport chain during diabetes [21]. On the other hand, mitochondria are the primary targets for oxidative stress because they lack protection from histone and are incapable of performing DNA repair functions by themselves [22]. Our previous observations have shown that the number of damaged mitochondrion in kidney proximal tubules was significantly higher in *Ins2*^*Akita/+*^ mice with 50% of reduced *Lias* gene expression than *Lias*^*+/+*^*Ins2*^*Akita/+*^ littermate controls. In the current studies, we consolidate our previous observation that mitochondrial damage in proximal tubules is a predominant pathological feature of DN by demonstrating that reduction in *Lias* gene expression in *Lias*^*Low/Low*^*Ins2*^*Akita/+*^ mice and increased *Lias* expression in *Lias*^*High/High*^*Ins2*^*Akita/+*^ mice impact mitochondrial integrity and function. Through use of *in vitro* LIAS knockdown studies it has been reported that ROS may decrease the mitochondrial membrane potential [23]. Given that mitochondrial dysfunction is postulated to be an initiator for diabetic complications [24], we presume that attenuation of DN by endogenous LA is likely, through preservation of mitochondrial function, to mediate reduction in mitochondrial ROS and mitochondrial damage. Thus, strengthening mitochondrial antioxidant capacity could be an effective means for prevention and treatment of DN. Our results are similar to a recent report indicating that *Ins2*^*Akita/+*^ mice specifically overexpressing catalase, a key antioxidant enzyme in renal proximal tubular cells, had reduced renal oxidative stress and attenuated progression of DN without changing blood glucose concentration [25]. Our contention, that mitochondrial damage mediates DN and that lipoic acid mitigates this injury, is further supported by another recent report that *Ins2*^*Akita/+*^ derived β-cells have increased mitochondrial dysfunction, oxidative stress, mitochondrial DNA damage, and alterations in mitochondrial protein levels that contribute to β-cell dysfunction [26].

LIAS synthesizes lipoic acid in mitochondria. Lipoic acid has a high reductive capacity and actively participates in the recycling of vitamin C and E. Diabetes mellitus is characterized by increased oxidative stress that negatively impacts mitochondrial integrity and function. Hence the physiologic importance of LIAS in diabetes is through its role in lipoic acid synthesis; lipoic acid may play a vital role in mitochondrial protection from oxidative stress and thus maintain energy balance during diabetes. Our previous data obtained from in *Lias*^*+/-*^*Ins2*^*Akita/+*^ mice showed that systemic and urinary oxidative stress markers significantly increased, whereas endogenous antioxidant capacity significantly decreased, when *Lias* gene expression levels was approximately 50%. Furthermore, reduced *Lias* gene expression in *Lias*^*+/-*^*Ins2*^*Akita/+*^ mice was associated with more severe DN pathological changes compared with *Lias*^*+/+*^*Ins2*^*Akita/+*^ mice. In particular, a large number of damaged mitochondria were detected in mouse proximal tubule epithelial cells as a unique phenomenon. The results observed in *Lias*^*Low/Low*^*Ins2*^*Akita/+*^ mice have confirmed that reduced *Lias* gene expression leads to decreased endogenous antioxidant capacity and mitochondrial damage in the proximal tubules. On the other hand, in *Lias*^*High/High*^*Ins2*^*Akita/+*^ mice harboring increased *Lias* gene expression, endogenous oxidant capacity and mitochondrial integrity are protected. Our data also indicate that kidney proximal tubules can serve as a window via which alternation of mitochondrial status due to oxidative stress can be assessed in diabetic nephropathy.

Based on ours and, other investigator’s data [23], we propose that several mechanisms are involved in the changes of kidney *Lias* expression and LA levels under diabetic conditions:

1. Excess ROS generated in diabetic mellitus impairs the antioxidant defense system, indicated by decline of reduced GSH levels, leading to further accumulation of ROS. The latter is reflected by a significantly enhanced lipid peroxidation levels in body and urine. Our new animal model with low expression of *Lias* gene further highlights this relationship between endogenous antioxidant levels, ROS concentrations and DN pathologic changes. That is, low endogenous antioxidant capacity will result in high levels of ROS and more severe DN.
2. Excessive ROS in diabetes mellitus damages mitochondria. Mitochondria are a major site of ROS generation and are vulnerable targets for ROS; hence, accumulated ROS due to insufficient LA protection in *Lias*^*Low/Low*^*Ins2*^*Akita/+*^ mice could damage mitochondria. We found that significant mitochondrial damage in *Lias*^*Low/Low*^*Ins2*^*Akita/+*^ mice; other investigators have also demonstrated decreased mitochondrial membrane potential using *in vitro* LASY knockdown method [23].

To investigate the mechanisms through which lipoic acid protects mitochondria in kidney proximal tubules, we examined gene expression of several common antioxidant enzymes in kidney cortex, including Sod series and glutathione peroxidase. Our data, using these novel mouse models, identify Sod2 as an antioxidant target by showing that expression of *Sod2 (*a major antioxidant enzyme which responds to enhanced superoxide production in mitochondria) markedly increases in kidney cortex of *Lias*^*Low/Low*^*Ins2*^*Akita/+*^ mice; the latter observation has been previously reported in the *Lias*^*+/-*^*Ins2*^*Akita/+*^ diabetic mice with 50% reduced LIAS [20]. SOD2 co-exists with Lias in mitochondria and is a major antioxidant enzyme that responds to enhanced superoxide production in mitochondria; hence, the observation that SOD2 increases in kidney cortex of *Lias*^*Low/Low*^*Ins2*^*Akita/+*^ mice with increased ROS is not surprising.

We attempted to identify antioxidant targets of LA in the diabetic kidney cortex. In addition to dysfunctional mitochondria, one of the most prominent sources of ROS is from the NADPH oxidase (NOX) activity [27–29]. Amongst these, the *Nox4* gene, a biomarker of oxidative stress in the diabetic mellitus, has particularly high expression in the kidney [30]. Our result showed that *Nox4* gene expression was significantly increased in *Lias*^*Low/Low*^*Ins2*^*Akita/+*^ and decreased in *Lias*^*High/High*^*Ins2*^*Akita/+*^ kidney cortex, respectively, suggesting that NADPH oxidase 4 is an antioxidant target of lipoic acid.

NRF2 is a key transcription factor for regulation of antioxidant defense [31]. Although it has been reported that LA stimulated NRF2 nuclear accumulation [32], our result do not detect any significant changes of *Nrf2* gene expression in 28-week-old *Lias*^*Low/Low*^*Ins2*^*Akita/+*^ and *Lias*^*High/High*^*Ins2*^*Akita/+*^ mouse kidney. Hence, the renal protective action mediated by increased LIAS-generated LA is not accompanied by *Nrf2* transcription alternation.

Several studies have revealed increased expression of TGF-β1 in renal glomeruli in experimental models of diabetes [34]. TGF-β1 is a critical mediator of podocyte injury and kidney hypertrophy characteristic of DN [33]. Thus, significantly reduced *Tgfβ1* gene expression in *Lias*^*High/High*^*Ins2*^*Akita*^ mice may play a role in attenuation of the adverse effect of diabetes mellitus on kidney structure and function. In addition, our results obtained from previous and the current studies using different degrees of *Lias* deficiency mouse models have clearly demonstrated that renal proximal tubules, with numerous mitochondria required for their important absorption functions, are sensitive to mitochondrion damage and that they are an ideal region of the nephron for assessment of mitochondria status during development of diabetic nephropathy. It is also worth studying the impact of mitochondrial damage on resorption in order to ascertain which mitochondrial component(s) is/are injured and the mechanism through which the deficiency of endogenous antioxidant protection develops.

Accumulating evidence support a role for inflammation in DN. In particular, the inflammatory cells infiltrate (like increased renal macrophage infiltration) and significantly higher levels of cytokines (chemokines) including monocyte chemoattractant protein-1 (MCP-1), accompanies DN [35]. MCP-1 is considered as a major mediator of inflammation process in DN patients [36]. It is a member of the CC chemokine family synthesized by a variety of cell types including glomerular endothelial cells, mesangial cells, tubular epithelial cells, and monocytes [37]. Studies suggest that MCP-1 production in mesangial cells and renal tubular epithelial cells are induced by advanced glycated end products (AGEs) through NF-κB activation [38, 39]. The promoters of MCP-1 gene contain binding sites for NF-kB [40]. Since urinary MCP-1 is upregulated in many renal diseases, including DN patients, we measured urinary MCP-1 levels in the three groups of mice. Our results showed that there were significantly increased urinary MCP-1 levels in *Lias*^*Low/Low*^*Ins2*^*Akita/+*^ mice compared with *Lias*^*+/+*^*Ins2*^*Akita/+*^ and *Lias*^*High/High*^*Ins2*^*Akita/+*^ mice. This observation indicates that increased inflammation occurs in *Lias*^*Low/Low*^*Ins2*^*Akita/+*^ mice. The increased MCP-1 is likely mediated by NF-kB in response to enhanced kidney oxidative stress [41, 42]. This result provides further evidence that increasing the endogenous antioxidant capacity could be an effective strategy for prevention and treatment of DN.

In a previous study, we showed that plasma glucose levels in *Lias*^*+/-*^*Ins2*^*Akita/+*^ mice are significantly greater than those in *Lias*^+/+^*Ins2*^*Akita/+*^ mice [20]. However, we did not detect any significant differences among the three groups of mice in the current study. We believe genetic background of the mice plays a major role in this discrepancy [43, 44]. We used mice with B6 genetic background for the current project, whereas we used F1 genetic background for the previous one. Thus, it is likely that even the same level of *Lias* gene expression in the different genetic background could exhibit different traits.

In summary, our data clearly indicate that *Lias*^*High/High*^*Ins2*^*Akita/+*^ and *Lias*^*Low/Low*^*Ins2*^*Akita/+*^ mice manifest variations in levels of endogenous antioxidant capacity in kidneys that lead to different degrees of diabetic pathologic changes. The results have clarified the role of antioxidants in the early development of diabetic nephropathy and strongly suggest that protection of mitochondria is a novel therapeutic target for effective antioxidant therapy of DN.

We conclude that these new antioxidant mouse models are suitable to elucidate the contributions of oxidative stress in the pathogenesis of diabetic kidney disease.

## Abbreviations

DN: diabetic nephropathy
Lias: LA, α-lipoic acid; lipoic acid synthase gene
3’-UTR: 3’-untranslated region
ROS: reactive oxygen species
MME: mesangial matrix expansion
4-HNE: 4-Hydroxynonenal
Sod2: superoxide dismutase 2
Tgfβ1: transforming growth factor β1
Nrf2: nuclear factor (erythroid-derived 2)-like 2
Nox4: NADPH oxidase 4
